# Impact of elexacaftor-tezacaftor-ivacaftor in lung transplantation for cystic fibrosis in the United States

**DOI:** 10.1016/j.jhlto.2024.100171

**Published:** 2024-10-26

**Authors:** Tahuanty A. Pena, Brittany Wright, Kalpaj R. Parekh, Julia Kleney-Tait

**Affiliations:** a Department of Internal Medicine, Stead Family Department of Pediatrics, University of Iowa Carver College of Medicine, University of Iowa Healthcare, Iowa City, Iowa; b Department of Pharmacy, University of Iowa Healthcare, Iowa City, Iowa; c Department of Cardiothoracic Surgery, University of Iowa Carver College of Medicine, University of Iowa Healthcare, Iowa City, Iowa; d Department of Internal Medicine, University of Iowa Carver College of Medicine, University of Iowa Healthcare, Iowa City, Iowa

**Keywords:** elexacaftor, tezacaftor, ivacaftor, cystic fibrosis, lung transplant

## Abstract

**Background:**

Cystic fibrosis (CF) is an autosomal recessive condition leading to progressive lung disease and often necessitating lung transplantation. Historically, CF has been one of the leading indications for lung transplants in the United States. The advent of CF transmembrane conductance regulator (CFTR) modulators, particularly elexacaftor-tezacaftor-ivacaftor (ETI), has significantly improved clinical outcomes for people with CF (pwCF), offering potential alterations in disease progression and transplantation needs.

**Methods:**

Data on lung transplants performed in the United States since 1988 were retrieved from the Organ Procurement & Transplantation Network. Custom reports were generated to compare the number of lung transplants and waitlist additions before and after ETI approval in 2019. The analysis focused on trends from 2009-2019 (pre-ETI) and 2021-2023 (post-ETI).

**Results:**

The average annual lung transplants for CF decreased significantly from 243 (2009-2019) to 56.7 (2021-2023) post-ETI approval. Similarly, the average number of pwCF added to the lung transplant waitlist per year dropped from 295 to 55.6. Despite an overall increase in lung transplants and waitlist additions in the United States, the proportion involving pwCF has markedly declined post-ETI.

**Conclusions:**

The introduction of ETI has dramatically reduced the need for lung transplants among pwCF, reflecting significant improvements in lung function and disease management. These findings underscore the transformative impact of CFTR modulators like ETI on the natural history of CF, highlighting the importance of continued advancements in precision medicine for genetic disorders. Future studies should investigate long-term outcomes and sustained trends in lung transplantation needs among pwCF.

## Background

Cystic fibrosis (CF) is an autosomal recessive condition that affects the CF transmembrane conductance regulator (CFTR). This defect results in abnormal ion transport, resulting in airway surface dehydration, airway obstruction, chronic infections, and inflammation.^1^ These processes frequently have an early onset in life and manifest clinically in early childhood,^2^ including the development of bronchiectasis with progressive loss of lung function, ultimately leading to advanced lung disease and lung failure.^3^

Traditionally, the care of CF lung disease has involved multiple measures to address the consequences of CFTR dysfunction. These include mucolytic therapies such as dornase alfa, mucus hydrating interventions with nebulized hypertonic saline, inhaled antibiotics, anti-inflammatories, and aggressive airway clearance measures.^4^ These measures improved median survival from early childhood in the 1950s to nearly 50 years of age by 2000.^5^

Lung transplantation is a treatment modality for people with advanced lung disease who fail to respond to available medical therapies and have limited short-term survival expectations.^6^ Historically, people with CF (pwCF) have represented the third most common reason for transplant in the United States, following interstitial lung disease and chronic obstructive pulmonary disease. Despite this, pwCF who have undergone lung transplants have better outcomes and lead long-term survival post-transplant compared to other diagnostic groups.^7^

In the last decade, the introduction of new therapeutics called CFTR modulators has revolutionized CF care. Four products have been approved by the Food and Drug Administration in the United States, starting with ivacaftor in 2012,^8^ followed by lumacaftor-ivacaftor in 2015,^9^ tezacaftor-ivacaftor in 2018,^10^ and most recently elexacaftor/tezacaftor/ivacaftor (ETI) in 2019.^11^ ETI showed superior outcomes in efficacy, even when compared to prior CFTR modulators, but also a greater potential treatment population, with approximately 94% of pwCF have an eligible genotype. The use of these therapies represents the first time we have been able to treat the underlying defect in the dysfunctional CFTR for those with applicable mutations as opposed to the downstream symptoms.

Initial research development and clinical trials of CFTR modulators did not include much representation from people with advanced lung disease or those who required transplantation. However, after the Food and Drug Administration approval, reports of using ivacaftor in people with advanced lung disease described appreciable benefits without significant added risks.^12^ Similar findings were described with newer generations of CFTR modulators, with more recent clinical trials including a wider representative population of people with advanced lung disease. Overall, these results suggest that people with advanced lung disease in CF had significant improvement in lung function and a significant reduction in pulmonary exacerbations, similar to those with better lung function.^13^

Since the approval of ETI in 2019, there have been significant improvements in CF lung disease in the clinical setting. We aimed to further evaluate the effect of ETI specifically on pwCF and their need for transplantation before and after 2020, when ETI was available for most patients.

## Materials and methods

We accessed data for transplants performed in the United States since 1988, which is publicly available through the Organ Procurement & Transplantation Network. Custom reports were built through their interactive website to obtain information regarding the number of lung transplants performed per year, indications, as well as the number of people added to the transplant waitlist. Reports were created through the Organ Procurement & Transplantation Network website (https://optn.transplant.hrsa.gov/data/view-data-reports/build-advanced/) between May 1 and 15, 2024. Patients with a heart-lung transplant or lung retransplantation were excluded. We looked at data for the prior 10 years before approval of ETI and compared it with the data available since. This work was exempt from Institutional Review Board review, as data are publicly available on the website.

## Results

There is an upward trend in the number of lung transplants in the United States each year, with 2023 surpassing 3,000 transplants. The average number of lung transplants performed per year over the 10 years preceding ETI approval (2009-2019) was 2085. In the 3 years following approval and assumed initiation of ETI for most pwCF (2021-2023), the average number of lung transplants increased to 2,747 per year.

**Figure 1:**
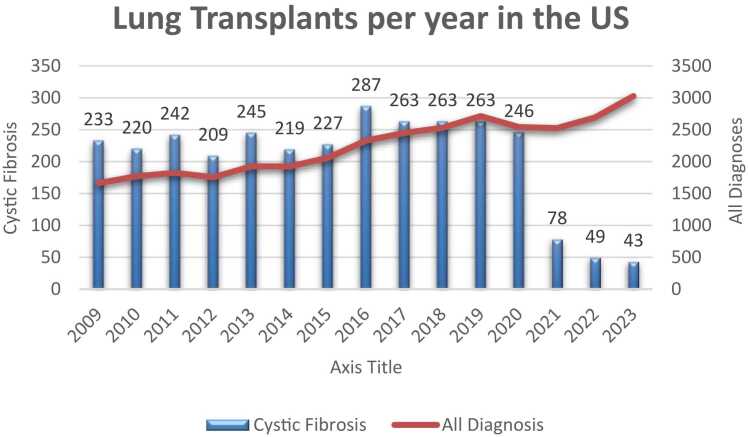
Lung transplants performed in the United States for all diagnoses and cystic fibrosis.

When the indication for transplant was defined as CF, between 2009 and 2019, there was an average of 243 transplants per year. In the period from 2021-2023, the average number of transplants per year due to CF decreased to just 56.7 (Figure 1).

**Figure 2:**
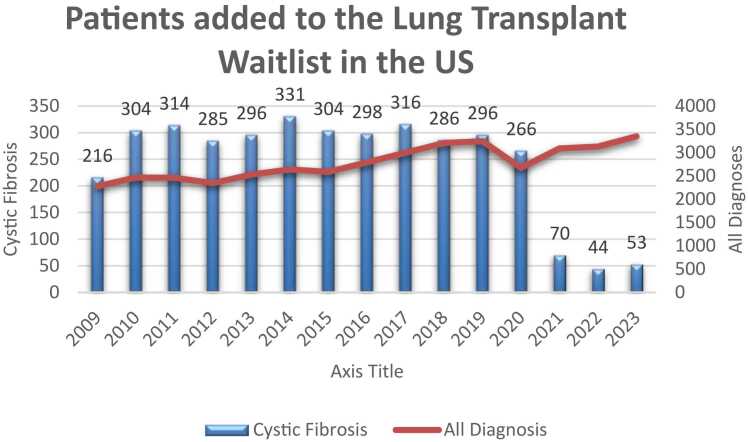
Patients added to the lung transplant waitlist every year in the United States.

The number of patients added to the lung transplant waitlist per year was also assessed. There were, on average, 2,687 patients added to the lung transplant list annually between 2009 and 2019. Of these individuals, 295 were pwCF. In the period from 2021 to 2023, there was an average of 3,191 total patients added per year, this included an average of only 55.6 pwCF per year (Figure 2).

## Discussion

The findings of this study reveal a significant shift in the landscape of lung transplantation for pwCF in the United States, coinciding with the approval and widespread adoption of ETI therapy. While the overall number of lung transplants in the United States continues to rise—surpassing 3,000 in 2023—the introduction of ETI in 2019 has profoundly impacted the need for transplantation among pwCF.

The decline in lung transplants for pwCF suggests that ETI may have played an important role in improving CF lung health, potentially contributing to fewer individuals reaching advanced stages of lung disease that would necessitate transplantation. Although the overall number of patients added to the transplant waitlist has grown, the significant reduction in pwCF listed further reinforces ETI’s positive clinical impact. Notably, in 2020, 36 pwCF were removed from the transplant waitlist due to “improved condition”—the highest number reported for this reason in over a decade. Despite these promising trends, the ongoing need for lung transplantation remains, driven by other conditions.

It is important to acknowledge several uncertainties moving forward. Patients who began CFTR modulator therapy after developing advanced lung disease may still experience disease progression, ultimately reaching end-stage lung disease and requiring transplantation. Additionally, lung transplantation will likely remain necessary for pwCF with genetic variants that do not respond to current CFTR modulators. These patients may represent a different subset compared to those who receive modulator treatment from an early age or earlier stages of disease progression, further reshaping the patient population requiring transplantation.

This study is subject to limitations, including its reliance on publicly available data, which may not fully capture the nuances of patient selection or clinical decision-making. Furthermore, the relatively short follow-up period since ETI’s introduction limits the ability to assess long-term transplantation trends in pwCF.

In conclusion, the sharp reduction in lung transplants and the declining number of pwCF added to the waitlist underscore the transformative impact of ETI. This CFTR modulator has not only improved the quality of life and survival outcomes for many pwCF but has also significantly altered the trajectory of advanced lung disease in CF. These findings highlight the importance of CFTR modulators in reshaping the natural history of CF and emphasize the need for continued advancements in precision medicine. Future research should focus on evaluating the long-term outcomes of ETI therapy, particularly for patients who began treatment with advanced lung disease, as well as the sustained impact on transplantation trends. Additionally, efforts must continue to develop therapies for those with mutation variants that are unresponsive to current modulators, ensuring that all pwCF have access to effective treatment options.

## Author contributions

T.P.: Conceived, designed, and performed analysis; data collection; manuscript writing and editing. B.W.: Manuscript writing and editing. K.P.: Manuscript writing and editing. J.K.T.: Manuscript writing and editing.

## Disclosure statement

The authors declare no personal or financial interests that could inappropriately influence or bias our work related to this article.

Acknowledgments: None.

Funding: There are no financial conflicts of interest to disclose by any of the authors.
